# Comprehensive analysis of two potential novel SARS-CoV-2 entries, TMPRSS2 and IFITM3, in healthy individuals and cancer patients

**DOI:** 10.7150/ijbs.51234

**Published:** 2020-09-30

**Authors:** Yu-Jun Dai, Wei-Na Zhang, Wei-Da Wang, Si-Yuan He, Cheng-Cai Liang, Da-Wei Wang

**Affiliations:** 1 Collaborative Innovation Center for Cancer Medicine, 651 Dongfeng East Road, Guangzhou, 510060, China.; 2 National Research Center for Translational Medicine, Ruijin Hospital affiliated to Shanghai Jiao Tong University School of Medicine, 197, Ruijin Road II, Shanghai, 200025, China.; 3 Department of Hematology, Guangzhou Women and Children's Medical Center, Guangzhou Medical University, Guangzhou, 510623, China.; 4 The University of Texas MD Anderson Cancer Center UTHealth Graduate School of Biomedical Sciences, Houston, TX 77030, USA.; 5 State Key Laboratory of Oncology in South China, Guangzhou, 510060, China.; 6 Department of Hematologic Oncology, Sun Yat-sen University Cancer Center, Guangzhou, 510060, China.

**Keywords:** SARS-CoV-2, Cancer, ACE2, TMPRSS2, IFITM3

## Abstract

Coronavirus disease 2019 (COVID-19) caused by SARS-CoV-2, with acute respiratory failure as the most significant symptom, has led to a global pandemic. Angiotensin-converting enzyme 2 (ACE2) is considered as the most important receptor of SARS-CoV-2 and wildly expressed in human tissues. Whereas, the extremely low expression of ACE2 in lung could hardly interpret the severe symptom of pneumonia in COVID-19 patients. Here we profiled two SARS-CoV-2 infection related genes, the transmembrane serine protease 2 (TMPRSS2) and the interferon-inducible transmembrane protein 3 (IFITM3), in human tissues and organs. Consistent with the expression and distribution of ACE2, TMPRSS2 was also highly expressed in digestive, urinary and reproductive systems, but low expressed in lung. Notably, the anti-virus protein IFITM3 also expressed much lower in lung than other tissues, which might be related to the severe lung symptoms of COVID-19. In addition, the low expression of IFITM3 in immune cells suggested that SARS-CoV-2 might attack lymphocytes and induce the cytokine release syndrome (CRS). Furthermore, cancer patients were considered as more susceptible to SARS-CoV-2 infection. Our data supposed that fourteen types of tumors might have different susceptibility to the virus according to ACE2, TMPRSS2 and IFITM3 expression patterns. Interestingly the prognosis of six types of cancers including breast carcinoma (BRCA), lung adenocarcinoma (LUAD), uterine corpus endometrial carcinoma (UCEC), renal clear cell carcinoma (KIRC), prostate adenocarcinoma (PRAD), and hepatocellular carcinoma (LIHC) were closely related to these gene expressions. Our study explored the expression and distribution profiles of two potential novel molecules that might participate in SARS-CoV-2 infection and involved in immunity, which may provide a functional basis for preventing infection of SARS-CoV-2.

## Introduction

A novel coronavirus (also known as severe acute respiratory syndrome coronavirus 2 (SARS-CoV-2)) broke out in Wuhan, China, in December 2019, which threatened global human health 1-4. It was also known as severe acute respiratory syndrome coronavirus (2019-nCoV) 5. The similarity between SARS-CoV-2 and SARS-COV is 82%, and the homology among them and the Middle East respiratory syndrome coronavirus (MERS COV) is 50% 6. Severe patients with COVID-19 may die from a large number of alveolar injuries and progressive respiratory failure and the mortality rate is approximately 3% 7, 8. Researchers have discovered that the novel coronavirus 2019, like SARS-COV, is also able to enter cells by using of S protein, which binds angiotensin-converting enzyme 2 (ACE2) on the surface of human cells 9, 10. Sequence comparison showed that SARS virus and ACE2 receptor combined with several key amino acids, which were highly conserved in the new coronavirus 11, 12. Since then, the S protein of the new coronavirus and ACE2 on the cell surface had become the research focus of scientists 13. ACE2 receptor is considered to be the key component of SARS-CoV-2 infecting human and spreading in host 14. In contrast, inconsistent with its severe lung symptoms, the ACE2 expression level in lung was very low. Therefore, we hypothesized that there might be new molecules recognized or regulated by the SARS-CoV-2.

Recently, Stefan Pöhlmann group revealed another key protein (transmembrane protease serine 2, TMPRSS2) was needed by the new coronavirus to invade cells. TMPRSS2 and endosomal cathepsin CatB can synergistically activate SARS-Cov-2 invading cells, while protease TMPRSS2 plays a more important role 15. In addition, interferon-induced transmembrane protein 3 (IFITM3) was involved in the process of cell differentiation, apoptosis, cell adhesion and immune cell regulation, which could help to enhance the immunity to influenza A (H1N1), West Nile virus and Dengue virus 16-18. Some researchers proved the association between IFITM and SARS-COV in 2011 19. Another group showed that IFITM protein restricted the infection mediated by Marburg virus and Ebola virus (Marv, EBOV) into glycoprotein 20. The researchers further demonstrated that the replication of SARS-COV and the invasion mediated by the spike protein of SARS-COV were also limited by the IFITM protein 21. IFITM limited the entry of a variety of envelope viruses into the host and regulated cell tropism independently of the expression of viral receptors 22.

Patients with cancers were considered to be more susceptible to SARS-CoV-2 infection and indicated poor prognosis 23. Here, we performed a profiling analysis on expression level of a novel SARS-CoV-2 receptor TMPRSS2 and an anti-virus protein IFITM in healthy individuals and patients with pan-cancers. This might improve the understanding on the type of cancers susceptible to virus and provide a functional basis for potential treatment of SARS-CoV-2.

## Materials and Methods

### The tissue and blood atlas database

The Human Atlas database 24 (https://www.proteinatlas.org/) contained large number of RNA and protein expression profiles in specific tissues and organs. It was mainly composed of 6 parts: tissue atlas, cell atlas, pathology atlas, brain atlas, blood atlas and metabolic atlas. The mRNA expression data from thirty-seven different normal tissues was derived from RNA-sequencing and the protein expression data was from protein profiling by immunohistochemistry. The Blood Atlas contained the transcriptomic sequencing data of more than fifteen types of immune cells including granulocytes, NK-cells, lymphocytes and hematopoietic stem/progenitor cells etc. 25. The RNA and protein expression levels of ACE2, TMPRSS2 and IFITM3 were analyzed by this database.

### ENCORI dataset

This dataset contained the gene expression data of more than 30 types of tumors 26. Pan-cancer analysis could be performed by researchers to explore the survival prognosis of ACE2, TMPRSS2 and IFITM3 and their differential expressed in cancers. The *P* value of differential expressed genes in all cancer was calculated through Student's *t* test and the survival data was analyzed by Kaplan-Meier and log-rank methods.

### Independent prognosis analysis tool

The biomedical database (http://bioinfo.henu.edu.cn/Index.html) focused on exploring the specific diagnostic and prognostic markers by using high-throughput sequencing data. The prognostic values of cancer subtypes were calculated by LOGpc system via TCGA data 27.

### The c-BioPortal analysis

Genetic landscape of mutations in ACE2, TMPRSS2 and IFITM3 were analyzed by the cBioCancer Genomics Portal (c-BioPortal, https://www.cbioportal.org/) based on The Cancer Genome Atlas (TCGA) 28, 29. The clinical data and genetic alternations were also provided by TCGA pan-cancer atlas studies. The mutation profiles and 3D structure of ACE2, TMPRSS2 and IFITM3 were also analyzed in mutation section of this database.

## Results

### Expression profile of TMPRSS2 in human tissues

**Figure 1:**
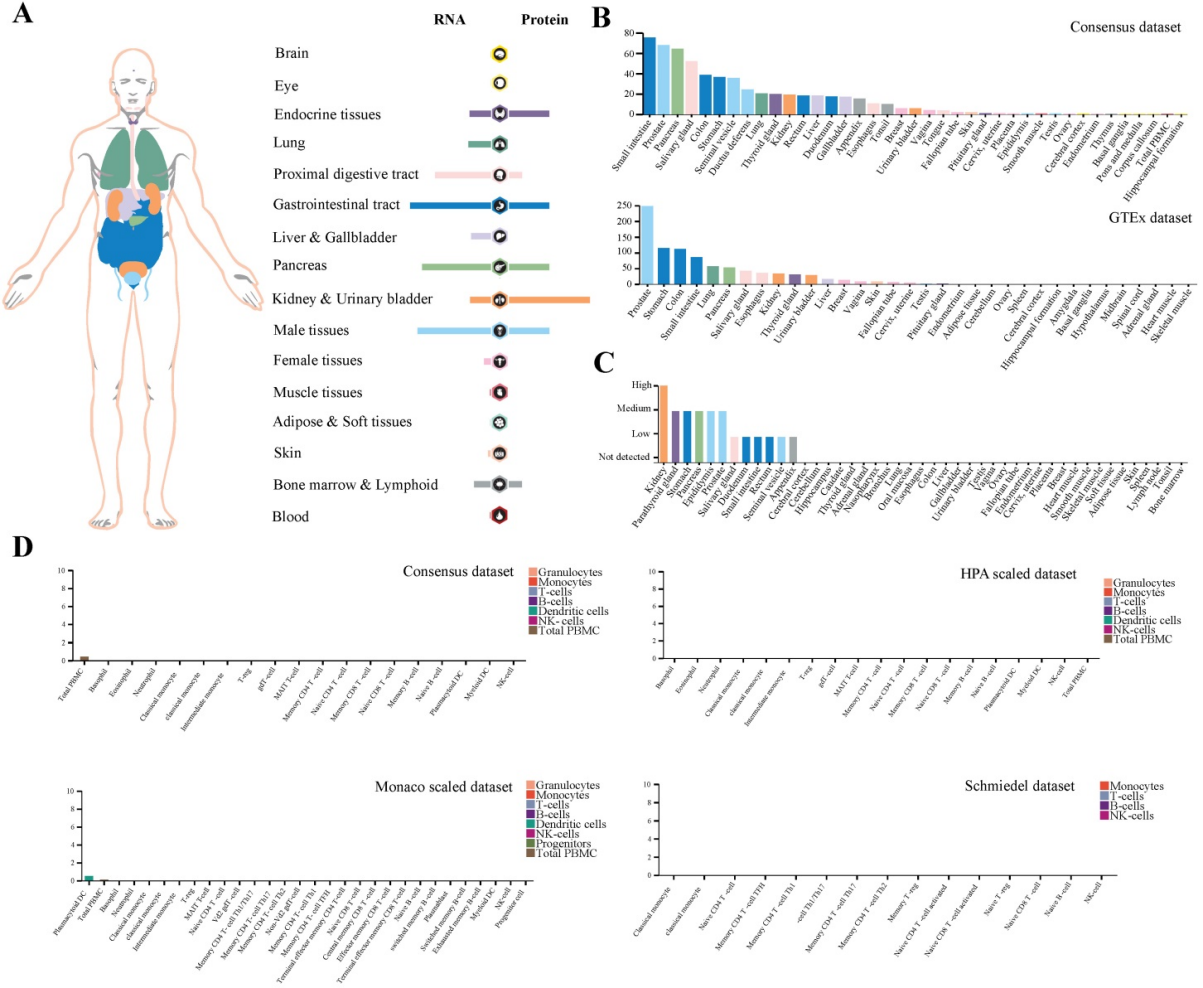
** Profiling of TMPRSS2 expressed in human tissues. (A)** The expression and distribution profile of TMPRSS2 in different tissues. **(B)** TMPRSS2 mRNA expression level of human tissues in Consensus dataset and GTEx dataset. **(C)** TMPRSS2 protein expression level of human tissues in related database. **(D)** Expression of TMPRSS2 in human blood cells.

Here, we demonstrated the expression profiling of TMPRSS2 at gene transcription and translation levels in human organs and tissues. We found that TMPRSS2 RNA was mainly expressed in proximal digestive tract, gastrointestinal tract, pancreas and male tissues. Whereas, the expression tendency of TMPRSS2 protein was different to its RNA and it was more expressed in endocrine tissues, gastrointestinal tract, kidney and urinary bladder and male tissues, which was consistent with the expression distribution of ACE2 protein at gene translation level (Figure 1A). Next, we further verified the expression of TMPRSS2 in Consensus dataset and GTEx dataset. The most expressed tissues of TMPRSS2 RNA were small intestine and prostate in Consensus dataset and GTEx dataset respectively. Both of these datasets showed that the RNA expression of TMPRSS2 was mainly distributed in gastrointestinal tract (including small intestine, colon and stomach), pancreas and prostate (Figure 1B). In addition, the protein expression dataset showed that TMPRSS2 protein was highly expressed in kidney, medium expressed in parathyroid gland, stomach, pancreas, epididymis and prostate and low expressed in salivary gland, duodenum, small intestine, rectum, seminal vesicle and appendix (Figure 1C). In accordance with the low expression level of ACE2 in lung and blood cells, TMPRSS2 also exhibited a relatively low expression status in lung especially at the protein level and it was also not detected in any type of immune cells in blood (Figure 1D).

### Expression profile of IFITM3 in human tissues

**Figure 2:**
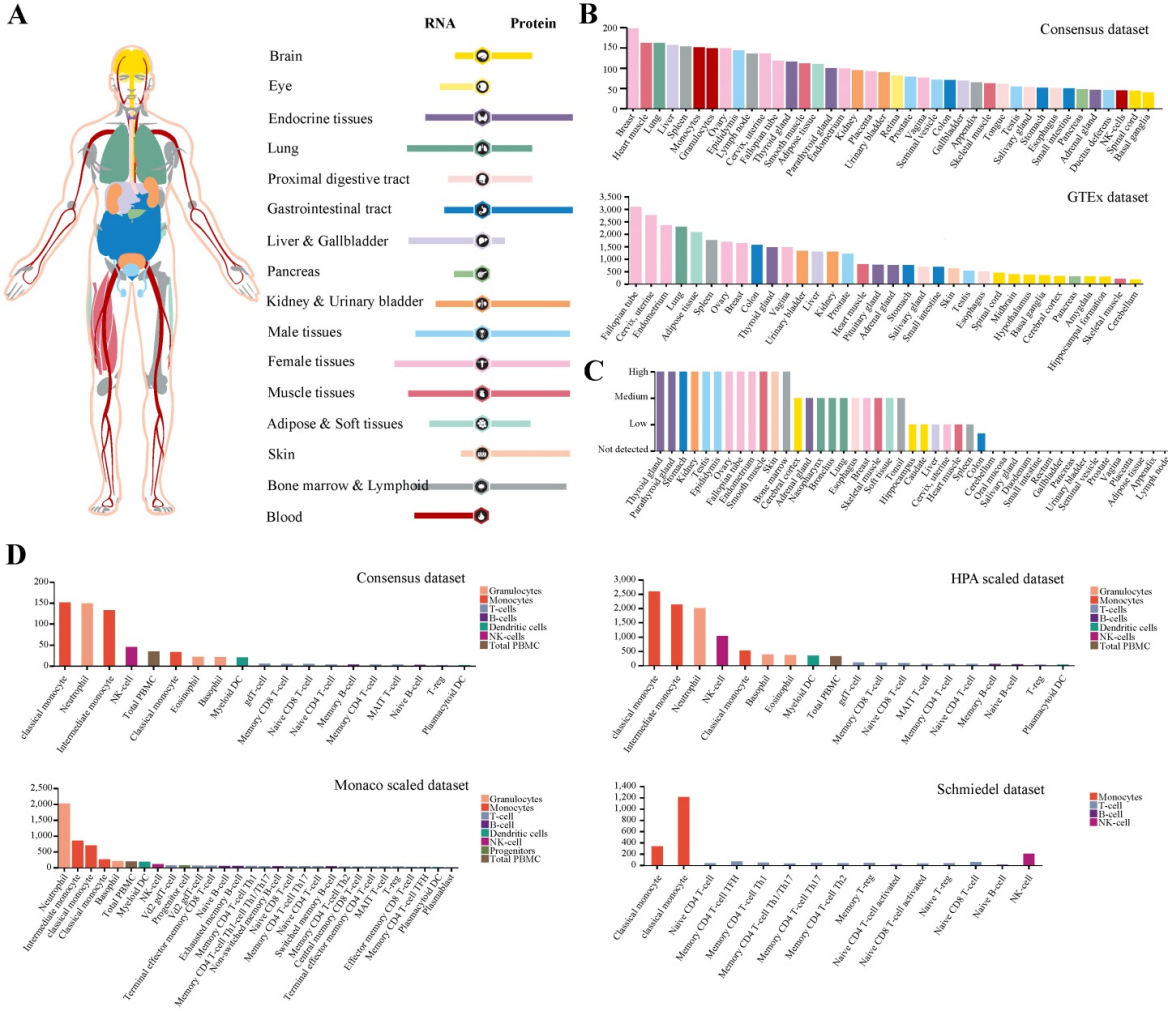
** Expression profile of IFITM3 in human tissues. (A)** The expression and distribution features of IFITM3 in human tissues. **(B-C)** IFITM3 expression level of human tissues at transcription level **(B)** and translation level **(C)**. **(D)** IFITM3 expression level of immune cells.

While the low expression of TMPRSS2 could not interpret SARS-CoV-2 infection in human, we noticed that the antivirus molecules may involve in its pathogenesis. Next, we paid more attention to protein IFITM3 and analyzed its expression and distribution in human tissues or organs (Figure 2A). The result demonstrated that IFITM3 was wildly expressed in all organs especially in lung, liver and gallbladder, female tissues, muscle tissues, bone marrow and lymphoid and blood cells at transcription level. The data was also confirmed by using Consensus dataset and GTEx dataset (Figure 2B). To our surprise, the IFITM3 protein expression level was totally different. At protein level, IFITM3 was more enriched in endocrine tissues, gastrointestinal tract, kidney and urinary bladder, male tissues and skin and the protein expression level was much more than that of RNA. In addition, lung, liver and gallbladder and blood cells were quite the contrary. It expressed much lower in these tissues compared with its RNA expression level (Figure 2A). We also validated this in protein expression dataset (Figure 2C). Furthermore, we explored the IFITM3 RNA expression in blood cells in Consensus dataset, HPA scaled dataset, Monaco scaled dataset and Schmiedel dataset. All the data in four datasets indicated that this gene was enriched in granulocytes and monocytes, but was hardly expressed in lymphocytes (T-, B- and NK-cells) in peripheral blood (Figure 2D).

### Expression profile of ACE2, TMPRSS2 and IFITM3 in pan-cancers

**Figure 3:**
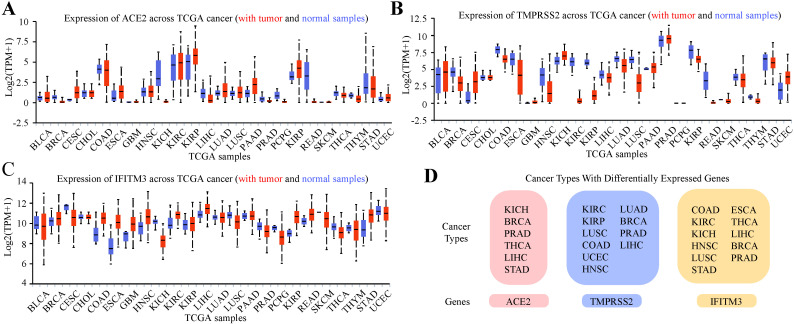
** Expression Profile of ACE2, TMPRSS2 and IFITM3 in Pan-cancers. (A-C)** Expression of ACE2 **(A)**, TMPRSS2 **(B)** and IFITM3 **(C)** across TCGA cancers (with tumor and normal samples). **(D)** The summary of tumor subtypes differentially expressed ACE2, TMPRSS2 and IFITM3.

Previous study indicated that tumor patients were more vulnerable to SARS-CoV-2 infection 23. We further analyzed the expression of the three genes in pan-cancers by using TCGA dataset and GTEx dataset (Figure 3A-C, Table S1-3). In total cancers, the cancer types with differentially expressed ACE2 were kidney chromophobe (KICH), breast invasive carcinoma (BRCA), prostate adenocarcinoma (PRAD), thyroid carcinoma (THCA), liver hepatocellular carcinoma (LIHC) and stomach adenocarcinoma (STAD). TMPRSS2 was differentially expressed in kidney renal clear cell carcinoma (KIRC), kidney renal papillary cell carcinoma (KIRP), lung squamous cell carcinoma (LUSC), colon adenocarcinoma (COAD), uterine corpus endometrial carcinoma (UCEC), head and neck squamous cell carcinoma (HNSC), lung adenocarcinoma (LUAD), BRCA, PRAD and LIHC and IFITM3 was differentially expressed in COAD, KIRC, KICH, HNSC, LUSC, STAD, esophageal carcinoma (ESCA), THCA, LIHC, BRCA and PRAD (Figure 3D).

### Mutation landscape of ACE2, TMPRSS2 and IFITM3 in pan-cancers

**Figure 4:**
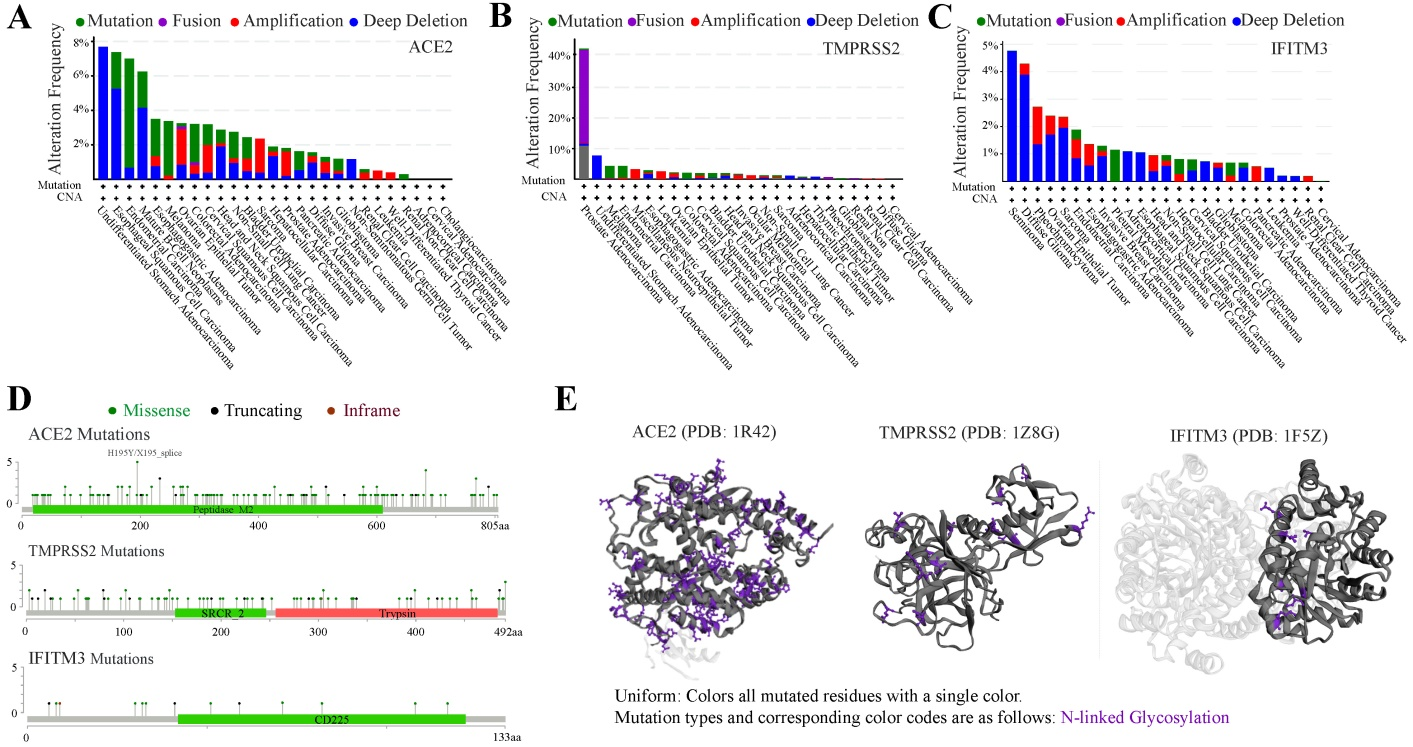
** Mutation spectrums of ACE2, TMPRSS2 and IFITM3 in cancers. (A-C)** The alteration frequency of mutations in ACE2 **(A)**, TMPRSS2 **(B)** and IFITM3 **(C)** based on TCGA dataset. **(D)** Natural variants of ACE2, TMPRSS2 and IFITM3 in cancers. **(E)** The 3D crystal structures of mutation and protein styles of ACE2 (PDB 1R42), TMPRSS2 (PDB 1Z8G) and IFITM3 (PDB 1F5Z).

Tumors were often accompanied by multiple gene mutations. We further analyzed the mutation status of ACE2, TMPRSS2 and IFITM3 in pan-cancers. The frequency of these genes mutated in different cancers was no more than 10%, except TMPRSS2 in prostate adenocarcinoma with nearly 40%. The top 3 cancers with mutated ACE2 were undifferentiated stomach adenocarcinoma, esophageal squamous cell carcinoma and endometrial carcinoma and those with TMPRSS2 were prostate adenocarcinoma, undifferentiated stomach adenocarcinoma and melanoma. While, IFITM3 was highly mutated in seminoma, diffuse glioma and pheochromocytoma (Figure 4A-C). The main mutation type of ACE2 and IFITM3 was deep deletion and that of TMPRSS2 was fusion. The detailed mutation landscapes of these three genes were exhibited in Figure 4D. Furthermore, 3D crystal structures of mutation and protein styles of ACE2 (PDB 1R42), TMPRSS2 (PDB 1Z8G) and IFITM3 (PDB 1F5Z) were analyzed in Figure 4E. Database results also showed that these mutated genes could up-regulate their expression levels in cancers. Next, we analyzed the relationship of mutation status among the three genes and we found that ACE2 could significantly coexist with TMPRSS2 and IFITM3 with *p* value 0.007 and 0.024, respectively. TMPRSS2 and IFITM3 mutations exhibited mutual exclusivity (Table S4).

### Prognosis of ACE2, TMPRSS2 and IFITM3 in pan-cancers

**Figure 5:**
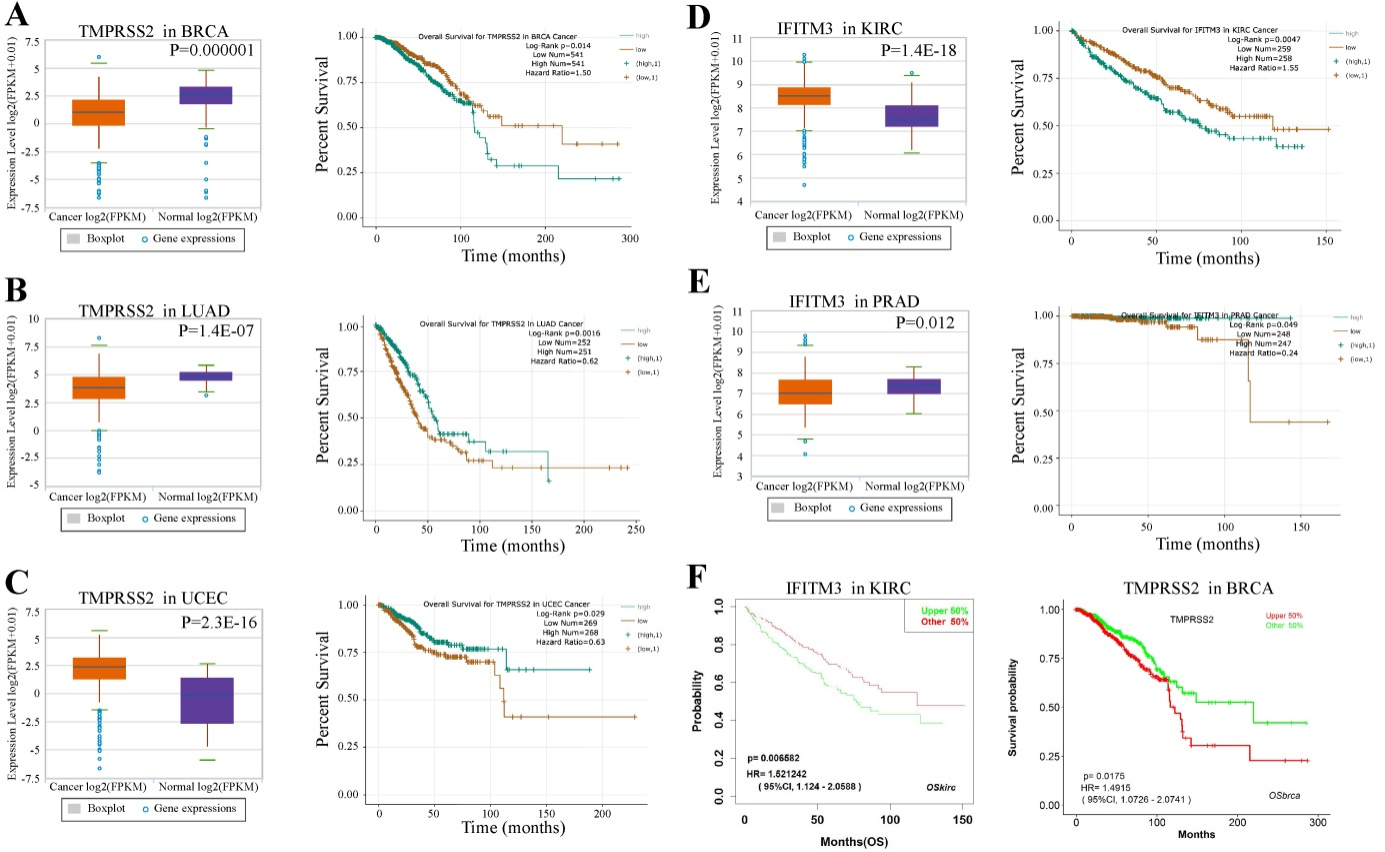
** Prognosis of TMPRSS2 and IFITM3 in cancers. (A-C)** The expression level and overall survival of TMPRSS2 in BRCA **(A)**, LUAD **(B)** and UCEC **(C)** compared with normal samples.** (D-E)** The expression level and overall survival of IFITM3 in KIRC **(D)** and PRAD **(E)**. **(F)** Prognosis analysis of TMPRSS2 and IFITM3 in different type of cancers by LOGpc system.

We next examined the prognostic survival of TMPRSS2 and IFITM3 in patients with cancers by ENCORI. We found that there were three cancer types were closely associated with TMPRSS2 and two types with IFITM3 (Table S5-7). More specifically, TMPRSS2 was expressed lower in breast cancer samples and lung adenocarcinoma than that in normal samples with *p* value 0.000001 and 1.4E-07, respectively. In contrast, the prognosis of TMPRSS2 was opposite. The high expression of TMPRSS2 might indicate a poor prognosis in BRCA (*p* = 0.014), but reveal a favorable prognosis in LUAD (*p* = 0.0016). In addition, the expression of TMPRSS2 was much higher in tumor cells of UCEC than that in normal samples (*p* = 2.3E-16) and the high expression of TMPRSS2 might suggest a favorable prognosis in UCEC (*p* = 0.029) (Figure 5A-C). About IFITM3, it was expressed higher in KIRC than that in healthy samples (*p* = 1.4e-18) and the high expression of IFITM3 might indicate a poor prognosis (*p* = 0.0047). Furthermore, the expression of IFITM3 was lower in samples of UCEC than that in controls (*p* = 0.012) and the high expression of IFITM3 might reflect a favorable prognosis in PRAD (*p* = 0.049) (Figure 5D-E). Moreover, we used an Independent Prognosis Analysis Tool to validate our findings by LOGpc system 27 as shown in Figure 5F and Figure S1.

## Discussion

At the end of 2019, a rapid outbreak of a new coronavirus (pathogen SARS-CoV-2) infected pneumonia swept the world. The novel coronavirus is mainly transmitted through respiratory droplets, digestive tract and contact 4, 12, 30, 31. Its evidence showing that in relatively closed environment, aerosol of certain concentration also has infectivity 32. Several studies have proved that ACE2 protein was wildly expressed in digestive, urinary and reproductive systems, which was highly consistent with the way the virus is transmitted and the clinical characteristics of patients infected with SARS-CoV-2 33-35. Multiple organ dysfunction syndrome (MODS) is the main cause of death triggered by COVID-19, of which acute respiratory failure is the most common 12. The deaths caused by COVID-19 pneumonia are mainly in patients over 65 years old, especially those with severe comorbidities. What caught our attention is that the expression of ACE2 was extremely low in lung cells, whereas the clinical symptoms of COVID-19 were most pronounced and fatal in lung 36.

Recently, researchers identified a novel receptor TMPRSS2 which could activate SARS-2-S invading cells synergy with ACE2 protein 15. And it has been reported that IFITM3 could inhibit the replication and invasion of SARS-COV 19. Thus, we intended to investigate whether the virus receptor TMPRSS2 and the anti-virus protein IFITM3 might be the potential entries of SARS-CoV-2. The expression and distribution of TMPRSS2 was highly expressed in endocrine tissues, gastrointestinal tract, kidney and urinary bladder and male tissues, which was consistent with those of ACE2 protein. This co-expression patterns further validated the SARS-CoV-2 entry process required the cell surface receptor ACE2 and TMPRSS2 37. In contrast, both of these two virus receptors were low expressed in lung cells. Unlike other viruses, the rapid overreaction of immune system is one of the important causes of death of SARS-CoV-2. This disparity might imply that host immune response could involve in the development and maintenance of pathological changes in COVID-19. To our surprise, we found the anti-virus protein IFITM3 was expressed much lower than other tissues and organs, which could explain the mechanism of viral pneumonia resulting in profound morbidity. Recent studies indicated that IFITM3 was upregulated explicitly in SARS-CoV-2 infected lung epithelial cells 38, 39. The genetic variants of IFITM3 were associated with disease severity in COVID-19 patients 40. Furthermore, our data also showed that in blood cells the IFITM3 was mainly expressed in granulocytes and monocytes, but no expressed in lymphocytes. In the lung tissue-resident memory CD8+ T-cells, IFITM3 expression could be up-regulated by antigen recognition or interferon-α to protect from viral infection 41. The innate immune response after initial SARS-CoV-2 infection might be malfunctioned characterized as delay or suppressed type I and III interferon response, suggesting that IFITM family members might be affected 16. Therefore, we wondered whether this phenomenon was associated with immune status of patients infected with SARS-CoV-2 and was related to the occurrence of cytokine storms. This still need further investigation.

Patients with tumors were considered to have become susceptible to SARS-CoV-2 23. The clinical features of cancer patients with COVID-19 are older, and the higher smoking history, the more the shortness of breath and more severe symptoms 7, 42. Once the cancer patients are infected with SARS-CoV-2, they easily progress to severe disease 43. Our result demonstrated that there were in total 14 types of tumors differentially expressed any of three proteins. Among them, we found that the types of tumors that differentially expressed IFITM3 are almost a combination of ACE2 and TMPRSS2. In addition, the categories between ACE2 and TMPRSS2 were also significant different and the difference in expression of all three proteins occurs only in three tumors (BRCA, PRAD and LIHC). Therefore, we suggested that IFITM3 could be used as an indicator of the susceptibility of SARS-CoV-2, not just ACE2 and TMPRSS2 in cancer patients. The profiling data on mutation status in ACE2, TMPRSS2 and IFITM3 indicated that low-frequency mutations may have little effect on the protein crystal structure and gene expression.

Previously we reported that liver cancer patients with high expression level of ACE2 should be more cautious to SARS-CoV-2 44. In this study, we explored two novel entries of SARS-CoV-2 in cancers and tried to expand tumor spectrum susceptible to the new coronavirus. Our result showed that fourteen types of cancers may have different susceptibility to SARS-CoV-2 and the expression level of the receptors or anti-virus proteins may affect the survival prognosis of patients with BRCA, LUAD, UCEC, KIRC, PRAD and LIHC. In UCEC and PRAD patients, the high expression of TMPRSS2 or low expression of IFITM3 in tumor tissues indicated a higher susceptibility to SARS-CoV-2 infection compared with the healthy individuals. Recent study demonstrated that male gender and the timepoint of cancer patients receiving chemotherapy before symptom onset were considered as risk factors for COVID-19 death during admission to hospital 45.

Taken together, the extremely low expression of ACE2 in lung may hardly justify the severity of pulmonary distress syndrome in SARS-CoV-2-infected patients. Our study is a proof of concept that new molecules recognized or regulated by the SARS-CoV-2, specifically TMPRSS2 and IFITM3 may be related to infection and anti-viral effect, respectively. Even though, implications of TMPRSS2 and of IFITM3 level of transcription and expression in different tissues/organs, and of differential expressions in different cancers, and specifically prognostic implications, are hypothesis-generating to be proven with further studies, our current research certainly would provide some very useful elements for understanding emerging pandemic pathogens.

## Supplementary Material

Supplementary figures and tables.

## Abbreviations

COVID-19: coronavirus disease 2019
SARS-CoV-2: severe acute respiratory syndrome coronavirus 2
MERS COV: middle east respiratory syndrome coronavirus
ACE2: angiotensin-converting enzyme 2
TMPRSS2: transmembrane serine protease 2
IFITM3: interferon-inducible transmembrane protein 3
CRS: cytokine release syndrome
Marv: marburg virus
EBOV: ebola virus
KICH: kidney chromophobe
BRCA: breast invasive carcinoma
PRAD: prostate adenocarcinoma
THCA: thyroid carcinoma
LIHC: liver hepatocellular carcinoma
STAD: stomach adenocarcinoma
KIRC: kidney renal clear cell carcinoma
KIRP: kidney renal papillary cell carcinoma
LUSC: lung squamous cell carcinoma
COAD: colon adenocarcinoma
UCEC: uterine corpus endometrial carcinoma
HNSC: head and neck squamous cell carcinoma
LUAD: lung adenocarcinoma
ESCA: esophageal carcinoma
MODS: multiple organ dysfunction syndrome
