# Supplementary material for: Comprehensive analysis of two potential novel SARS-CoV-2 entries, TMPRSS2 and IFITM3, in healthy individuals and cancer patients
Source: Int J Biol Sci. 2020 Sep 30;16(15):3028–36. doi: 10.7150/ijbs.51234 (PMC7545701; doi:10.7150/ijbs.51234)
Supplement: Supplementary file 1 — Supplementary figures and tables. [file ijbsv16p3028s1.pdf]

Supplemental Materials

Comprehensive Analysis of Two Potential Novel SARS-CoV-2 Entries, TMPRSS2 and IFITM3, in Healthy Individuals and Cancer Patients

Yu-Jun Dai[1,5,6,*,#], Wei-Na Zhang[3,*], Wei-Da Wang[1,5,6,*], Si-Yuan He[4,*], Cheng-Cai Liang[1,5], Da-Wei Wang[2,#]

1. Collaborative Innovation Center for Cancer Medicine, 651 Dongfeng East Road, Guangzhou, 510060, China

2. National Research Center for Translational Medicine, Ruijin Hospital affiliated to Shanghai Jiao Tong University School of Medicine, 197, Ruijin Road II, Shanghai, 200025 China

3. Department of Hematology, Guangzhou Women and Children's Medical Center, Guangzhou Medical University, Guangzhou, 510623, China

4. The University of Texas MD Anderson Cancer Center UTHealth Graduate School of Biomedical Sciences, Houston, TX 77030, USA

5. State Key Laboratory of Oncology in South China, Guangzhou, China

6. Department of Hematologic Oncology, Sun Yat-sen University Cancer Center, Guangzhou, China

SUPPLEMENTARY INFORMATION

Table S1. ACE2 significant differentially expressed in six type of cancers compared with normal samples.

Cancer	Cancer Full Name	Cancer Number	Normal Number	CancerExp	Normal Exp	Flod Change	p Value	FDR
KICH	Kidney Chromophobe	65	24	0.1	9.49	0.01	1.10E-27	1.50E-25
BRCA	Breast Invasive Carcinoma	1104	113	1.09	0.48	2.27	9.10E-22	8.20E-21
PRAD	Prostate Adenocarcinoma	499	52	0.19	1.8	0.11	1.60E-13	3.40E-12
THCA	Thyroid Carcinoma	510	58	0.6	0.72	0.83	0.00074	0.0023
LIHC	Liver Hepatocellular Carcinoma	374	50	2.34	1.07	2.19	0.0012	0.0038
STAD	Stomach Adenocarcinoma	375	32	4.24	35.62	0.12	0.0048	0.013
LUAD	Lung Adenocarcinoma	526	59	2.79	0.82	3.38	0.047	0.097
UCEC	Uterine Corpus Endometrial Carcinoma	548	35	0.66	0.38	1.75	0.039	0.1
KIRP	Kidney Renal Papillary Cell Carcinoma	289	32	40.82	20.93	1.95	0.057	0.14
COAD	Colon Adenocarcinoma	471	41	13.62	16.42	0.83	0.073	0.17
PAAD	Pancreatic Adenocarcinoma	178	4	4.83	0.78	6.18	0.046	0.38
ESCA	Esophageal Carcinoma	162	11	2.62	0.91	2.87	0.21	0.5
HNSC	Head and Neck Squamous Cell Carcinoma	502	44	1.41	1.1	1.17	0.47	0.62
CHOL	Cholangiocarcinoma	36	9	10.94	1.22	8.97	0.56	0.67
BLCA	Bladder Urothelial Carcinoma	411	19	1.28	1.01	1.26	0.48	0.73
KIRC	Kidney Renal Clear Cell Carcinoma	535	72	22.66	17.24	1.31	0.72	0.76
LUSC	Lung Squamous Cell Carcinoma	501	49	1.56	0.84	1.85	0.67	0.77

Table S2. TMPRSS2 significant differentially expressed in ten type of cancers compared with normal samples.

Cancer	Cancer Full Name	Cancer Number	Normal Number	CancerExp	Normal Exp	Flod Change	p Value	FDR
KIRC	Kidney Renal Clear Cell Carcinoma	535	72	1.47	21.24	0.07	1.4E-90	3.3E-88
KIRP	Kidney Renal Papillary Cell Carcinoma	289	32	1.89	21.19	0.09	1.1E-38	1.3E-36
LUSC	Lung Squamous Cell Carcinoma	501	49	4.56	27.8	0.16	2.2E-32	4.3E-31
COAD	Colon Adenocarcinoma	471	41	34.3	80.75	0.42	1.2E-23	3.4E-22
UCEC	Uterine Corpus Endometrial Carcinoma	548	35	6.36	1.58	4.03	2.3E-16	4.3E-15
HNSC	Head and Neck Squamous Cell Carcinoma	502	44	2.39	8.73	0.27	6.1E-13	1.1E-11
LUAD	Lung Adenocarcinoma	526	59	20.02	29.42	0.68	1.4E-07	7.4E-07
BRCA	Breast Invasive Carcinoma	1104	113	3.19	7.48	0.43	0.000001	0.0000037
PRAD	Prostate Adenocarcinoma	499	52	251.1	184.9	1.36	0.000012	0.000074
LIHC	Liver Hepatocellular Carcinoma	374	50	5.77	9.02	0.64	0.0001	0.00039
KICH	Kidney Chromophobe	65	24	51.51	25.05	2.06	0.027	0.063
ESCA	Esophageal Carcinoma	162	11	14.99	36.17	0.41	0.013	0.072
STAD	Stomach Adenocarcinoma	375	32	22.61	27.85	0.81	0.055	0.1
THCA	Thyroid Carcinoma	510	58	4.7	4.04	1.17	0.11	0.21
PAAD	Pancreatic Adenocarcinoma	178	4	14.62	8.04	1.82	0.02	0.24
BLCA	Bladder Urothelial Carcinoma	411	19	16.85	8.09	2.08	0.14	0.34
CHOL	Cholangiocarcinoma	36	9	15.47	8.42	1.84	0.72	0.75

Table S3. IFITM3 significant differentially expressed in 11 type of cancers compared with normal samples.

Cancer	Cancer Full Name	Cancer Number	Normal Number	CancerExp	Normal Exp	Flod Change	p Value	FDR
COAD	Colon Adenocarcinoma	471	41	403.13	136.06	2.96	2.40E-21	5.50E-20
KIRC	Kidney Renal Clear Cell Carcinoma	535	72	384.87	216.88	1.77	1.40E-18	1.20E-17
KICH	Kidney Chromophobe	65	24	90.86	273.5	0.33	6.70E-15	1.20E-13
HNSC	Head and Neck Squamous Cell Carcinoma	502	44	444.93	194.49	2.29	4.60E-11	6.10E-10
LUSC	Lung Squamous Cell Carcinoma	501	49	276.15	412.79	0.67	3.70E-08	1.60E-07
STAD	Stomach Adenocarcinoma	375	32	490.32	239.77	2.04	2.20E-08	2.00E-07
ESCA	Esophageal Carcinoma	162	11	318.69	123.37	2.58	1.40E-08	8.10E-07
THCA	Thyroid Carcinoma	510	58	129.34	164.32	0.79	2.60E-05	0.00011
LIHC	Liver Hepatocellular Carcinoma	374	50	795.2	532.06	1.49	0.00026	0.00089
BRCA	Breast Invasive Carcinoma	1104	113	349.27	246.43	1.42	0.0045	0.01
PRAD	Prostate Adenocarcinoma	499	52	157.68	172.69	0.91	0.012	0.038
CHOL	Cholangiocarcinoma	36	9	568	388.22	1.46	0.17	0.33
UCEC	Uterine Corpus Endometrial Carcinoma	548	35	607.77	587.71	1.03	0.18	0.34
KIRP	Kidney Renal Papillary Cell Carcinoma	289	32	268.14	202.85	1.32	0.2	0.39
LUAD	Lung Adenocarcinoma	526	59	351.86	333.02	1.06	0.33	0.46
BLCA	Bladder Urothelial Carcinoma	411	19	319.44	248.74	1.28	0.53	0.77
PAAD	Pancreatic Adenocarcinoma	178	4	409.69	376.13	1.09	0.9	0.95

Table S4. The relationship of mutation status among ACE2, TMPRSS2 and IFITM3 in cancers.

A	B	Neither	A Not B	B Not A	Both	Log2 Odds Ratio	p-Value	q-Value	Tendency
ACE2	TMPRSS2	9596	228	348	17	1.04	0.007	0.021	Co-occurrence
ACE2	IFITM3	9833	238	111	7	1.382	0.024	0.035	Co-occurrence
TMPRSS2	IFITM3	9707	364	117	1	-2.133	0.072	0.072	Mutual exclusivity

Table S5. The efficiency of ACE2 expression in the survival of pan-cancer patients.

Cancer	Cancer Full Name	Cancer Number	Median	coef.	HR	p Value
KIRC	Kidney Renal Clear Cell Carcinoma	517	15.45	-0.59	0.55	0.00016
MESO	Mesothelioma	85	0.03	-0.73	0.48	0.0028
OV	Ovarian Serous Cystadenocarcinoma	374	0.15	-0.36	0.7	0.0073
LIHC	Liver Hepatocellular Carcinoma	369	0.22	-0.43	0.65	0.017
LUSC	Lung Squamous Cell Carcinoma	469	0.8	-0.28	0.76	0.054
STAD	Stomach Adenocarcinoma	365	1.28	-0.06	0.94	0.7
KICH	Kidney Chromophobe	64	0.07	-0.16	0.86	0.82
LUAD	Lung Adenocarcinoma	503	1.02	0.03	1.03	0.84
BRCA	Breast Invasive Carcinoma	1082	0.05	-0.03	0.97	0.85
PRAD	Prostate Adenocarcinoma	495	0.1	-0.02	0.98	0.97

Table S6. The efficiency of TMPRSS2 expression in the survival of pan-cancer patients.

Cancer	Cancer Full Name	Cancer Number	Median	coef.	HR	p Value
LUAD	Lung Adenocarcinoma	503	14.97	-0.48	0.62	0.0016
BRCA	Breast Invasive Carcinoma	1082	2.11	0.41	1.5	0.014
UCEC	Uterine Corpus Endometrial Carcinoma	537	5.33	-0.47	0.63	0.029
UVM	Uveal Melanoma	80	0.01	0.9	2.45	0.052
KICH	Kidney Chromophobe	64	43.86	-1.27	0.28	0.09
BLCA	Bladder Urothelial Carcinoma	406	8.53	-0.24	0.78	0.11
THYM	Thymoma	118	0.12	-1.09	0.34	0.13
SARC	Sarcoma	261	0.01	0.27	1.31	0.18
DLBC	Lymphoid Neoplasm Diffuse Large B-cell Lymphoma	47	0.01	-1.04	0.35	0.19
READ	Rectum Adenocarcinoma	159	30.36	0.49	1.64	0.22

Table S7. The efficiency of IFITM3 expression in the survival of pan-cancer patients.

Cancer	Cancer Full Name	Cancer Number	Median	coef.	HR	p Value
LGG	Brain Lower Grade Glioma	523	61.96	1.01	2.76	2.2E-08
SKCM	Skin Cutaneous Melanoma	440	289.56	-0.42	0.66	0.0023
UVM	Uveal Melanoma	80	359.91	1.36	3.9	0.0031
KIRC	Kidney Renal Clear Cell Carcinoma	517	367.53	0.44	1.55	0.0047
MESO	Mesothelioma	85	902.49	-0.68	0.51	0.0058
SARC	Sarcoma	261	352.69	-0.53	0.59	0.0098
ACC	Adrenocortical Carcinoma	79	133.74	0.87	2.38	0.024
PRAD	Prostate Adenocarcinoma	495	130.26	-1.43	0.24	0.049
TGCT	Testicular Germ Cell Tumors	139	276.22	20.21	596651275.6	0.083
HNSC	Head and Neck Squamous Cell Carcinoma	495	334.72	0.21	1.24	0.12
BRCA	Breast Invasive Carcinoma	1082	277.42	-0.21	0.81	0.19
THYM	Thymoma	118	173.27	0.79	2.21	0.25
LUSC	Lung Squamous Cell Carcinoma	469	236.4	0.16	1.17	0.26
LAML	Acute Myeloid Leukemia	75	22.94	0.32	1.38	0.28
OV	Ovarian Serous Cystadenocarcinoma	374	938.85	-0.14	0.87	0.28
PCPG	Pheochromocytoma and Paraganglioma	183	83.28	-0.73	0.48	0.32
LIHC	Liver Hepatocellular Carcinoma	369	722.18	0.17	1.19	0.33
BLCA	Bladder Urothelial Carcinoma	406	195.33	0.15	1.16	0.34
PAAD	Pancreatic Adenocarcinoma	178	373.1	0.19	1.2	0.38
KICH	Kidney Chromophobe	64	79.61	-0.6	0.55	0.39

Figure S1

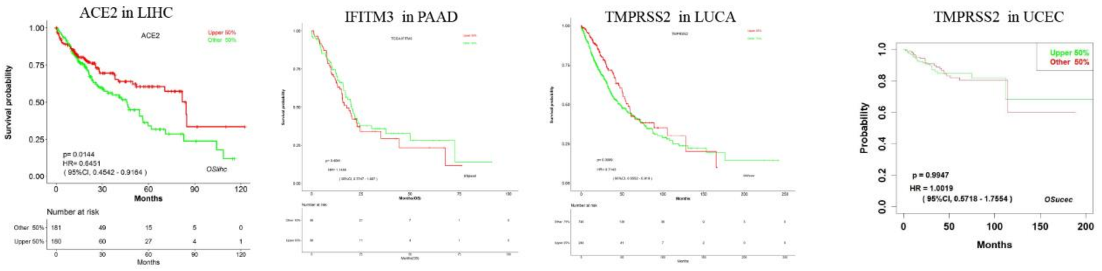

Figure S1. Prognosis analysis of ACE2, TMPRSS2 and IFITM3 in cancers by LOGpc system.
